# Supplementary material for: Modulation of Antioxidant Enzyme Expression of In Vitro Culture-Derived Reticulocytes
Source: Antioxidants (Basel). 2024 Sep 2;13(9):1070. doi: 10.3390/antiox13091070 (PMC11429491; doi:10.3390/antiox13091070)
Supplement: Supplementary file 1 [file antioxidants-13-01070-s001.zip › antioxidants-3124836-supplementary.pdf]

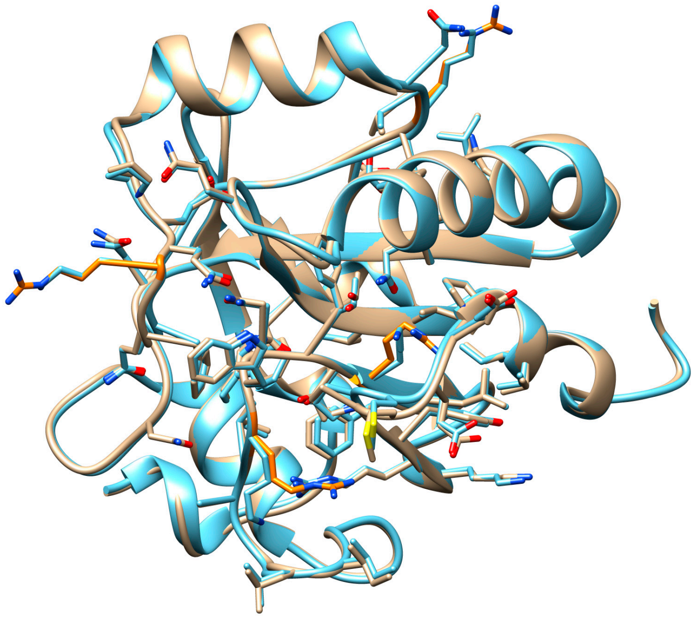

Supplemental Figure S1 – Modelled ribbon structure of human GPx4 with mutated ubiquitination sites. Blue ribbon and sticks represents the structure of wildtype human GPx4 based on 7L8Q.pdb. This is overlayed with that of a minimised model whereby lysine ubiquitination sites are mutated to arginines (wheat ribbon, arginines have orange carbons).
